# Quantitative proteomics of HFD-induced fatty liver uncovers novel transcription factors of lipid metabolism

**DOI:** 10.7150/ijbs.71431

**Published:** 2022-05-01

**Authors:** Shang Zhi, Zhang Congcong, Gao Zhiling, Qian Yihan, Xin Yijing, Liu Guanjie, Wang Fang, Sun Xuehua, Li Hongjie, Kong Xiaoni, Gao Yueqiu

**Affiliations:** 1 Central Laboratory, Department of Liver Diseases, ShuGuang Hospital Affiliated to Shanghai University of Chinese Traditional Medicine, Shanghai, China; 2 Institute of Infectious Disease, ShuGuang Hospital Affiliated to Shanghai University of Chinese Traditional Medicine, Shanghai, China; 3 Department of Ultrosound, Shuguang Hospital Affiliated to Shanghai University of Chinese Traditional Medicine, Shanghai, China; 4 Department of General Surgery, Shanghai Ninth People' Hospital, Shanghai Jiao Tong University School of medicine, Shanghai, China

**Keywords:** quantitative proteomics, NAFLD, transcription factor, lipid metabolism

## Abstract

Nonalcoholic fatty liver disease (NAFLD) has become the most common chronic liver disease, which progression is tightly regulated by transcription factors (TFs), nuclear receptors, and cellular enzymes. In this study, a label-free quantitative proteomic approach was used to determine the effect of the high-fat diet on the proteomics profile of liver tissue and to identify novel NAFLD related TFs. Mice were fed with HFD for 16 weeks to establish a NAFLD mouse model. Mice fed with normal chow diet were taken as controls. Liver samples were collected from each group for proteomics analysis. A total of 2298 proteins were quantified, among which 106 proteins were downregulated, while 256 proteins were upregulated in HFD-fed mice compared with the controls with fold change more than 1.5 and p value less than 0.05. Bioinformatic analysis revealed that metabolic-related functions and pathways were most significantly enriched. A subgroup of 11 TFs were observed to share interactions with metabolic-related enzymes and kinases by protein-protein interaction analysis. Among them, 7 TFs were selected for verification, and 3 TFs were finally validated, including Rbbp4, Tcea1, and ILF2. Downregulating each of the 3 TFs could significantly promote lipid accumulation in AML12 hepatocytes, by regulating the expression of fatty acid synthesis- or β-oxidation-related genes. In contrast, overexpression of Tcea1, Rbbp4, and ILF2, respectively, could ameliorate hepatocyte steatosis. These findings propose novel lipid metabolism related TFs, which might have potential roles in preventing NAFLD.

## Introduction

Nonalcoholic fatty liver disease (NAFLD), a spectrum of liver disease characterized by fat accumulation, affects more than 25% of the global population 1. Hepatic steatosis has been recognized as a critical trigger for the more advanced disease, nonalcoholic steatohepatitis (NASH), which increases the incidence of liver cirrhosis and hepatocellular carcinoma 2-4. Despite considerable studies, the molecular mechanisms of NAFLD remain incompletely understood. Currently, no FDA-approved pharmacological strategy has been established for NAFLD 5. Thus, further studies are warranted to investigate novel therapeutic targets.

Lipids produced by *de novo* lipogenesis have been considered to be the fundamental step in the development of NAFLD and are caused by the imbalance between triglyceride (TG) synthesis and degradation 6. The dysregulation of key enzymes involved in fatty acid synthesis or oxidation, including ATP citrate lyase (ACLY), fatty acid synthase (FASN), acetyl-CoA (ACC), and carnitine palmitoyltransferase-1α (Cpt1α), leading to direct TG accumulation 7. Previous studies have revealed several protein subtypes that can regulate the expression or degradation of these enzymes, such as transcription factors (TFs)^8^, RNA binding proteins 9, and ubiquitin ligases 10. Among TFs, sterol regulatory element-binding transcription factor 1 (SREBP1) and peroxisome proliferator-activated receptors (PPARs), which have been well-studied, regulate the cellular level of lipogenic and fat-consuming enzymes by directly binding to their gene encoding elements 11, 12. Agents targeting SREBP1 and PPARs are undergoing clinical trials for NAFLD 13. Considering the crucial role of TFs, the development of new TFs involved in regulating NAFLD progression has attracted the attention of investigators.

In recent years, proteomics analysis based on high-throughput mass spectrometry (MS) has been used to study different aspects of NAFLD. Plasma proteome profiling studies have identified several potential biomarkers related to NAFLD stages 14. Quantitative proteomics has also been applied to uncover differential expressed proteins (DEPs) in NAFLD using clinical liver tissues or mouse models 15, 16. Although hundreds of DEPs have been identified, the precise link between DEPs and NAFLD settings is rarely verified. Furthermore, there are limited proteomics studies on NAFLD-related TFs screening.

In the present study, a label-free quantitative proteomics strategy was used to identify hepatic DEPs related to NAFLD, followed by the construction of protein interaction network and gene ontology enrichment through Ingenuity Pathway Analysis (IPA). Among hundreds of DEPs, TFs were screened out for in vivo and in vitro verification. The potential role of validated TFs including transcription elongation factor A protein 1 (Tcea1), histone-binding protein RBBP4 (Rbbp4), and interleukin enhancer-binding factor 2 (ILF2) was further investigated by gene knockdown and overexpression strategies.

## Materials and methods

### Animal experiments

6-week-old male C57BL/6 mice were obtained from the center of experimental animals of Shanghai University of Traditional Chinese Medicine and were housed in specific pathogen free conditions on a 12 h light/dark cycle with free access to water and food. The mice were set on the following diets: HFD mice fed with high-fat diet (Dyets, 112252, USA, containing 60% kcal fat, 20% kcal carbohydrate, and 20% protein) for 16 weeks to establish NAFLD mouse model; NCD mice were fed with normal chow diet for 16 weeks. Body weights of mice from both groups were recorded every week. The mice were sacrificed at the age of 22 weeks, and all procedures were conducted in compliance with institutional guidelines and protocols. Liver samples after perfusion were collected and stored in liquid nitrogen for histological evaluation and proteomics analysis. All animal experiments were performed with approval from the Institutional Animal Care and Use Committee of Shuguang Hospital, Shanghai Chinese traditional medicine University.

### Liver histological evaluation

Liver tissues were fixed overnight in 4% PFA in PBS at room temperature, embedded in paraffin, and then stained with hematoxylin and eosin (H&E) for assessment of liver histology. Liver steatosis was calculated by hepatocyte steatosis and ballooning, as described previously 17. Oil Red O staining was performed on the frozen sections to evaluate lipid accumulation in the liver lobes using standard methods 18.

### Blood parameters

The concentrations of the total cholesterol (TC), triglyceride (TG), low-density lipoprotein cholesterol (LDL-C), high-density lipoprotein cholesterol (HDL-C), hepatic enzymes alanine amino transferase (ALT), aspartate amino transferase (AST), and alkaline phosphatase (ALP) were measured using kits according to manufacturer's instructions and analyzed by using a spectrophotometer (SpectraMax iD5).

### Clinical nonalcoholic fatty liver and control liver samples

Clinical NAFLD and control liver tissues were obtained from liver biopsy of NAFLD patients. The severity of hepatic steatosis was diagnosed by abdominal ultrasound and was verified according to liver histology. These patients were admitted to the ShuGuang Hospital Affiliated to Shanghai University of Chinese Traditional Medicine. The study protocol was approved by the Ethics Committee of ShuGuang Hospital Affiliated to Shanghai University of Chinese Traditional Medicine (IRB 2020-878-87).

### Western blot

Equal amounts of proteins were separated on a 10% pre-cast SDS-PAGE (Genscript, Nanjing, China), and then transferred to polyvinylidene difluoride (PVDF) membranes (PALL Life Sciences, USA). The membranes were blocked with 5% milk in TBST (TBS with 0.1% Tween-20) for 1 h at room temperature, and then incubated overnight at 4 ℃ with primary antibodies: anti-Rbbp4 antibody (Proteintech, 20364-1-AP), anti-Tcea1 antibody (Proteintech, 17825-1-AP), anti-ILF2 antibody (Proteintech, 14714-1-AP), anti-NPM1 antibody (Proteintech, 10306-1-AP), anti-Acc1 antibody (abcam, ab45174), anti-Fasn antibody (abcam, ab128870), anti-Srebp1c antibody (abcam, ab28481), anti-Acox1 antibody (abcam, ab184032), anti-Cpt1α antibody (abcam, ab128568), anti-Flag antibody (Proteintech, 20543-1-AP), anti-GAPDH antibody (abcam, ab8245), anti-β-actin antibody (Proteintech, 66009-1-Ig). The membranes were washed and incubated with horseradish peroxidase-conjugated secondary antibodies for 1 h. Protein bands were visualized using enhanced chemiluminescence kit (UUbio, Suzhou, China) with a Tanon 5200 multi-imaging system (Tanon Technology Co., Ltd, Shanghai, China).

### Sample preparation for label-free proteomics

The total proteins were extracted from perfused liver tissues by homogenizing in RIPA lysis buffer (Thermo scientific, Waltham, USA). After centrifugation at 12,000g for 10 min at 4 °C, the supernatant was collected and the protein concentrations were determined using the BCA kit (UUbio, Suzhou, China), according to the manufacturer`s instructions. 100 μg of protein samples were reduced using 20 mM dithiothreitol (DTT, Sigma-Aldrich) at 60 °C for 60 min, and 100 mM iodoacetamide was used for alkylated at room temperature for 40 min. Alkylated protein was precipitated overnight at -20℃ by adding 5 times the volumes of acetone. The protein precipitates were resuspended in 100 mm ammonium bicarbonate, followed by digested overnight with trypsin at a trypsin-to-protein mass ratio of 1:50. Finally, the peptide was desalted using the MnoSpin C18 column (GL Sciences, Japan), vacuum-dried, reconstituted in 0.1% formic acid.

### LC-MS/MS analysis and bioinformatics analysis

The peptides were analyzed using LC-MS/MS on Q Exactive™ HF-X (Thermo Fisher Scientific) coupled to an EASY-nLC 1000 UPLC system (Thermo Fisher Scientific). MS1 was set at a resolution of 70,000 and then selected for MS/MS with an NCE setting of 28. The MS2 was set at a resolution of 17,500. Quantitative data analysis was performed by MaxQuant (v1.6.1.2) using raw data files of MS/MS spectra searched against the UniProtKB mouse database (release 2021_04, 17090 entries) concatenated with reverse decoy database. Trypsin/P was used as the cleavage enzyme allowing up to two missing cleavages. The mass tolerance for precursor ions in First search and in Main search was set as 20 ppm and 6 ppm, respectively, and for fragment ions was set to 0.02 Da. Carbamidomethylation of cysteines was defined as fixed modification, while protein N-terminal acetylation and methionine oxidation were defined as variable modifications for database searches. Maximum FDR, calculated by employing a reverse database strategy, was set to 1% for peptides and proteins. Label-free quantification was performed by Intensity-based absolute quantification (iBAQ). Protein expression from each sample was compared using normalized iBAQ value, based on at least two unique peptides, and data processing was aligned. The 1.5 folds cut-off was set up for the screening of differentially expressed proteins.

Gene ontology (GO) annotation, KEGG pathways were performed using Omicsolution online tool (https://omicsolution.org/wkomics/main/). P-value < 0.05 was considered to be statistically significant. Quantitative data of differentially expressed proteins was submitted to Ingenuity Pathway Analysis (IPA, QIAGEN, Redwood City, http://www.ingenuity.com/) for analysis of protein-protein interaction.

### Immunohistochemistry analysis

Formalin-fixed liver tissue samples were analyzed by immunohistochemistry. Briefly, mouse liver sections were deparaffinized, repaired with 0.01 M sodium citrate-hydrochloric acid buffer solution, and incubated with primary antibodies, including Rbbp4, Tcea1, and ILF2 (1:100 dilution) at 4 °C overnight. The incubated liver sections were treated with peroxidase-conjugated secondary antibody (111-035-003, Jackson ImmunoResearch, USA) at room temperature, stained with diaminobenzidine for 3-5 min, and counterstained with hematoxylin. The liver sections were then observed under an optical microscope.

### siRNA transfection and knockdown

AML12 and HepG2 cells were transfected using RNAiMAX transfection kits (Thermo scientific, USA) with negative control siRNA or siRNA oligonucleotides specific to Rbbp4, Tcea1, and ILF2 according to manufacturer's instructions. After 48 h of transfection, cells were collected and used for downstream applications. The siRNA sequences were listed in Table S1.

### Plasmid construction and transfection

Plasmids encoding full-length Rbbp4, Tcea1, and ILF2 were constructed by cloning the indicated coding regions of mouse Rbbp4 cDNA, Tcea1 cDNA, or ILF2 cDNA into the pEnCMV-Flag vector. AML12 cells were transfected using lipofectamine 3000 transfection kits (Thermo scientific, USA) with negative control plasmid or overexpression plasmid specific to Rbbp4, Tcea1, and ILF2 according to manufacturer's instructions. After 48 h of transfection, cells were collected and used for downstream applications.

### Statistical analysis

Data were expressed as mean ± standard deviation. Two tailed student's t test was used to analyze the differences between two groups. P value less than 0.05 was considered statistically significant. Statistical analysis was performed using Graph Pad Prism 8 (version 8.2.1).

## Results

### Construction and evaluation of steatohepatitis by high-fat diet in mice

**Figure 1:**
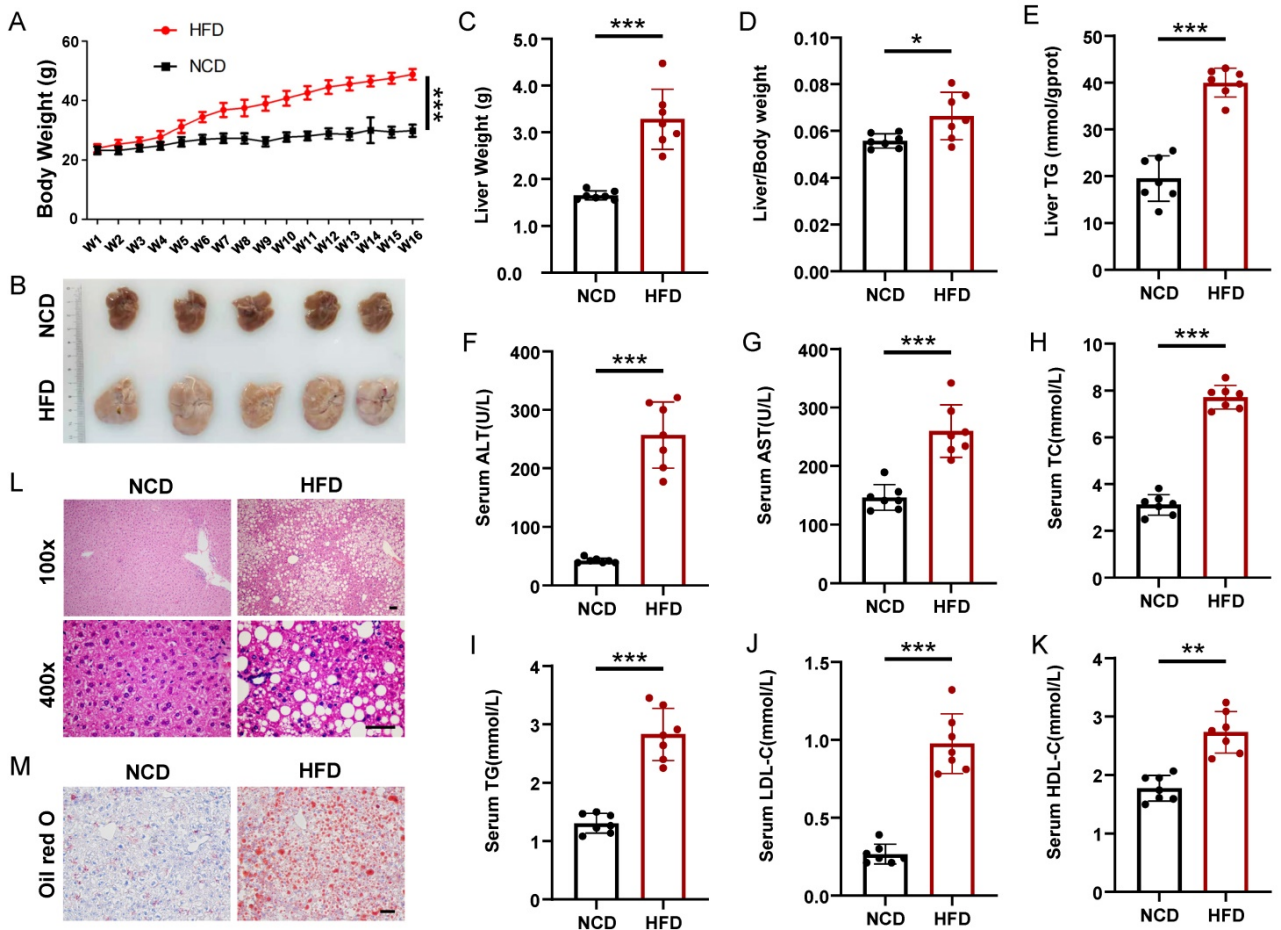
Mice model of high-fat diet induced nonalcoholic fatty liver. (A) Body weight gain of HFD- and NCD-fed mice (n=7 mice per group). (B) Gross morphology of livers from HFD- and NCD-fed mice. (C-K) Liver weight, liver/body weight ratio, liver TG, serum ALT, serum AST, serum TC, serum TG, serum LDL-C, and serum HDL-C of the HFD and NCD groups (n=7 mice per group). (L) H&E staining of livers from HFD- and NCD-fed mice. Magnification: 100x and 400x, scale bar: 50 μm. (M) Oil red O staining of livers from HFD- and NCD-fed mice. Scale bar: 50 μm. All data represent the mean ± SEM. Student's t test,* p < 0.05, ** p < 0.01, *** p < 0.001.

To induce hepatic steatosis, we fed mice a high-fat diet (HFD) for 16 weeks, whereas the control group maintained a normal chow diet (NCD). The body weight of the mice in the HFD group increased significantly compared with that in the NCD group, which was nearly two times that of the NCD group at the 16th week (Figure 1A). Firstly, we performed glucose tolerance test (GTT) and insulin tolerance test (ITT) on HFD and NCD-fed mice, HFD mice displayed enhanced glucose intolerance and insulin resistance compared to NCD mice (Figure S1). All mice were sacrificed 16 weeks after HFD. Liver weight and liver/body ratios of the HFD group were significantly higher than those of the NCD group (1.65 ± 0.09 g vs. 3.14 ± 0.65 g, n = 7, p < 0.001; 5.6 ± 0.28% vs. 6.7 ± 0.43%, n = 7, p < 0.05), and the fatty liver had almost 100% increase in hepatic triglyceride (TG) (Figure 1B-1E). What's more, the HFD group also displayed elevated serum levels of alanine transaminase (ALT), aspartate transaminase (AST), total cholesterol (TC), TG, LDL-C, and HDL-C compared with the NCD group (Figure 1F-1K). Liver HE staining results showed steatosis of hepatocytes, slight inflammatory cell infiltration, and ballooning degeneration in HFD-fed mice (Figure 1L). Excessive fat droplets accumulation in the hepatocytes of HFD-fed mice was then confirmed by the Oil red O staining (Figure 1M).

### Identification and quantification of differentially expressed proteins (DEPs) related to steatohepatitis

**Figure 2:**
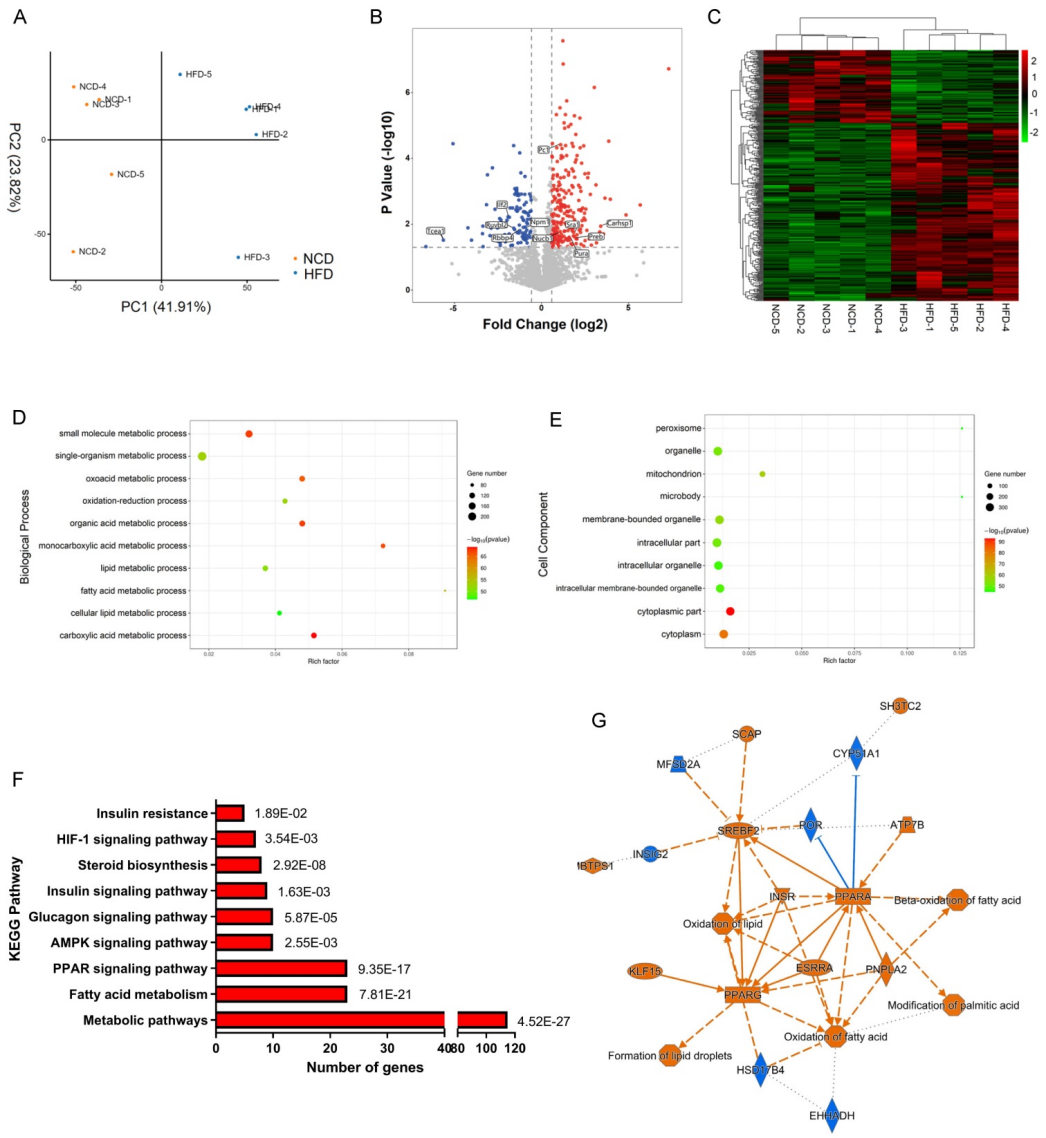
Label-free quantification proteomics of livers from HFD- and NCD-fed mice. (A) Principle component analysis (PCA) for proteome of all liver samples. (B) Volcano plot of DEPs. Red and blue dots represent upregulated and downregulated proteins, respectively. (C) Heatmap of DEPs. (D) Biological process enrichment of DEPs. (E) Cell component enrichment of DEPs. (F) KEGG pathway enrichment of DEPs. (G) DEPs-upstream regulators-biological processes interaction network constructed by IPA. Blue represents inhibition of protein/biological process, Orange represents activation of protein/biological process.

We then analyzed the total proteome alteration of these mice by label-free quantification based on LC-MS/MS. Equal amounts of liver proteins from the NCD and HFD groups were reduced, alkylated and digested with trypsin, with 5 biological replicates per group. Principle component analysis (PCA) of protein expression profiles of all samples showed that the global patterns of the proteome differed between the NCD and HFD groups (Figure 2A). However, individual differences were noted within each population. A total of 2298 proteins were identified with at least two unique peptides at a false discovery rate of < 1%. After quantification, 362 proteins were screened out as DEPs, with fold change more than 1.5 and p value less than 0.05, among which 106 DEPs were downregulated, while 256 DEPs were upregulated in HFD-fed mice compared with the controls (Table S2, Figure 2B), and the heatmap of DEPs was shown in Figure 2C.

### Gene ontology and KEGG pathway enrichment analysis of DEPs

Further bioinformatics analysis was performed on the 362 DEPs identified above. Among the altered proteins, 62.7% were annotated by Gene Ontology (GO) as involved in metabolic processes (Figure S2A). The highly enriched proteins were associated with lipid metabolic process, fatty acid metabolic process, organic acid metabolic process, and oxidation-reduction process (Figure 2D). As expected, DEPs were significantly enriched in mitochondrion, peroxisome, and microbody, where organelles metabolic catalysis took place (Figure 2E). KEGG pathway enrichment analysis demonstrated that metabolism-related pathways including PPAR signaling, AMPK signaling, insulin signaling, and fatty acid metabolic signaling were mainly enriched pathways (Figure 2F). Based on the DEPs, we constructed DEPs-upstream regulators-biological processes network by ingenious pathway analysis (IPA), showing that lipid oversupply could activate PPAR-α, PPAR-γ, SREBPs, ESRR, and INSR in hepatocytes, resulting in activation of fatty acid oxidation and lipid droplets formation (Figure 2G).

**Figure 3:**
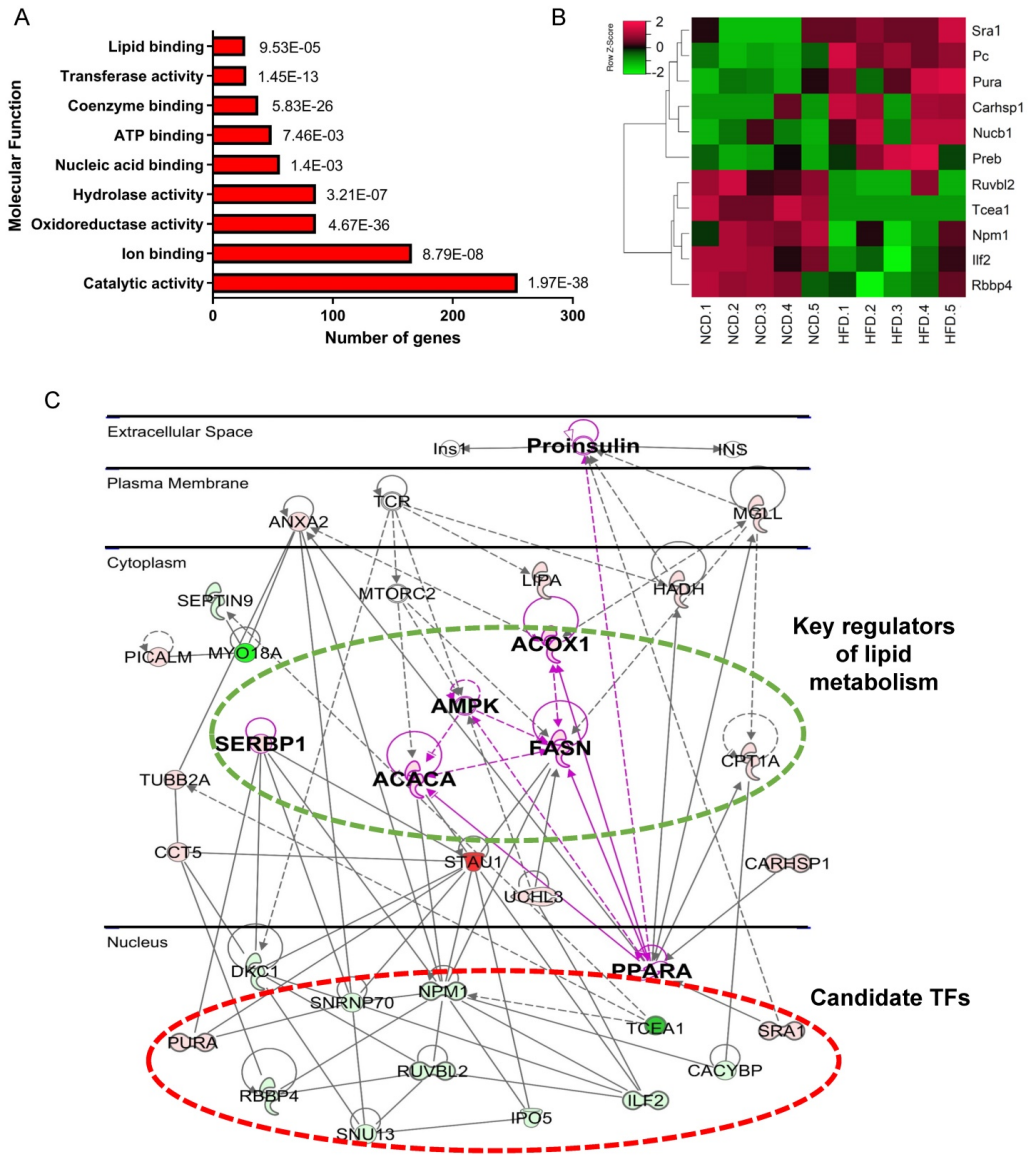
Bioinformatics analysis of DEPs revealed metabolic dysfunction related TFs. (A)Molecular function enrichment of DEPs. (B) Heatmap of differential abundance TFs. (C) Protein-protein interaction network contructed by IPA. Green represents downregulated DEPs, red represents upregulated DEPs, gray represents upstream reguators of DEPs.

In the molecular function category, 69% of DEPs were proteins with catalytic activity (Figure 3A), among which Acly, Acox1, Acc1, Hadha, and Acadm were top 5 high-weight catalytic enzymes in weight distribution analysis results (Figure S2B). Other highly enriched molecular functions included ion binding, ATP binding, and nucleic acid binding (Figure 3A), indicating that metabolism disturbance of hepatocytes was the major trigger of NAFLD.

### Screening and validation of candidate proteins in vivo and in vitro

We noticed that 56 DEPs had nucleic acid binding capacity, including transcription factors (TFs) and RNA binding proteins. Considering the crucial role of TFs in regulating gene expression and signaling transduction, 11 differential expressed TFs were selectited for further analysis (Figure 3B and Table 1). PPI network was then constructed using IPA, which revealed the interactions of these 11 TFs with key proteins in lipid metabolism, such as SREBP1, AMPK, PPARa, FASN, and Acc1 (Figure 3C). The result suggests that these 11 TFs may regulate genes implicated in fatty acid metabolism. Among the 11 TFs, Ruvbl2, Pc, Sra1, and Preb have been reported to regulate lipid metabolism 19-22, we therefore chose the remaining 7 TFs for further validation.

**Figure 4:**
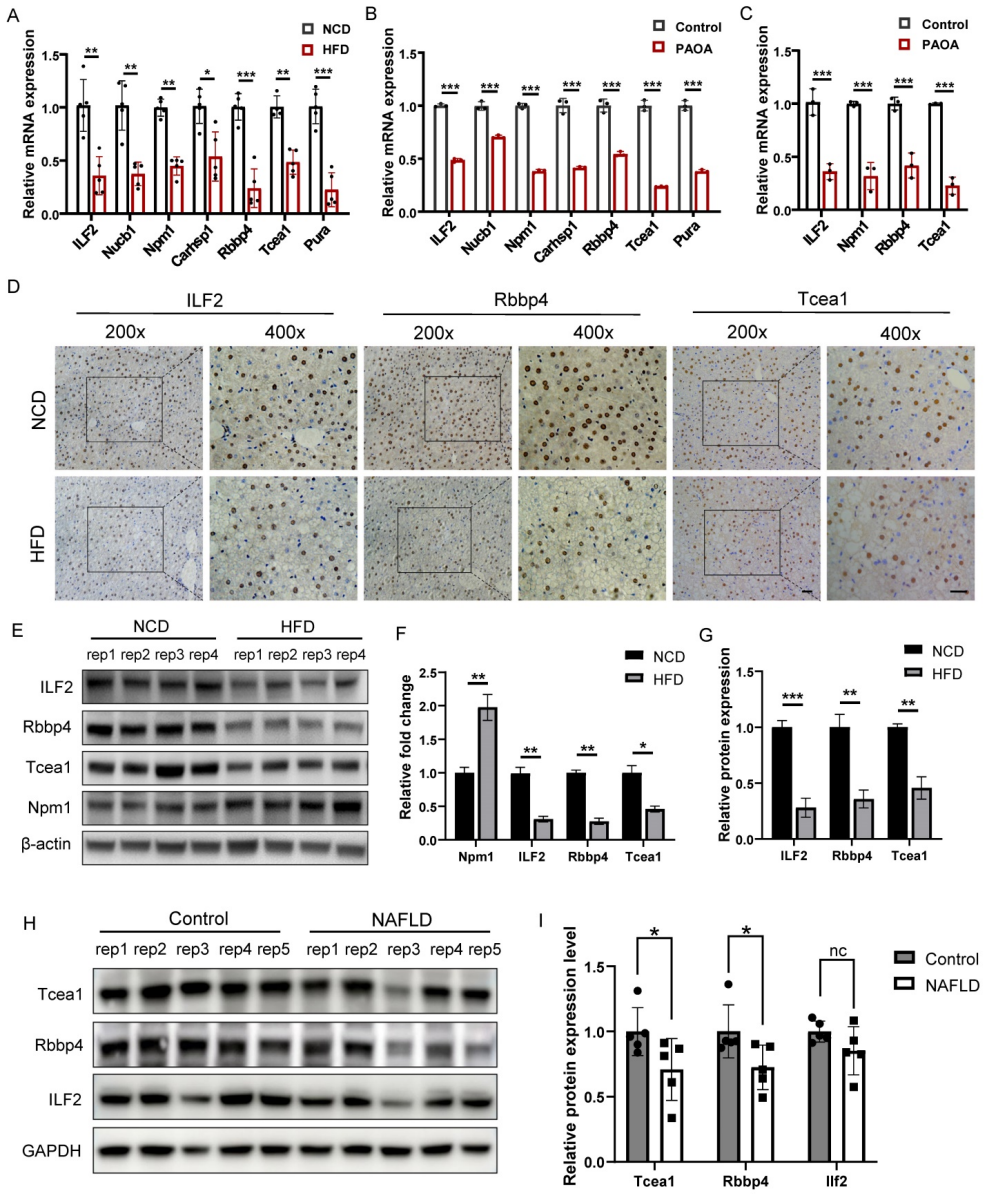
Verification of candidate TFs in vivo and in vitro. (A) Verification of candidate genes mRNA expression in liver tissues of HFD- and NCD-fed mice (n=5). (B) Verification of candidate genes mRNA expression in AML12 cells after PAOA treatment for 24 h (n=3). (C) Verification of candidate genes mRNA expression in primary hepatocytes after PAOA treatment for 24 h (n=3). (D) Representative images of immunohistochemistry analysis of Tcea1, Rbbp4, ILF2 expression and localization in liver sections from HFD- and NCD-fed mice. Magnification: 200x and 400x, scale bar: 50 μm. (E) Western blot analysis of ILF2, Rbbp4, Tcea1, and Npm1 expression in liver tissues from HFD- and NCD-fed mice. (F) Quantification of western blot analysis of ILF2, Rbbp4, Tcea1, and Npm1 protein expression (n=4). (G) Quantification of immunohistochemistry analysis of Tcea1, Rbbp4, and ILF2 expression (n=3). (H) Western blot analysis of ILF2, Rbbp4, and Tcea1 expression in liver tissues from NAFLD patients and control. (I) Quantification of western blot analysis of ILF2, Rbbp4, and Tcea1protein expression (n=5). All data represent the mean ± SEM. Student's t test, * P < 0.05, ** P < 0.01, *** P < 0.001.

We first verified the expression of 7 TFs at transcription level in liver tissues, which were significantly downregulated in the HFD group compared to the NCD group (Figure 4A). The same result was also observed in AML12 hepatocytes treated with palmitic acid/oleic acid (PAOA, 0.25 mM) for 24 h (Figure 4B). 4 TFs whose transcription level were consistent with the mass spectrometry result, including ILF2, Npm1, Rbbp4, and Tcea1, were chosen for validation in primary hepatocytes. PAOA treatment caused a significant decrease in the transcriptional expression of these 4 TFs in primary hepatocytes (Figure 4C). We then confirmed the changes in the protein levels of these 4 TFs in liver tissues by western blot, which revealed the downregulation of ILF2, Rbbp4, and Tcea1 in HFD-feeding mice (Figure 4E and 4F). In addition, immunohistochemical staining showed the same result, and expression of ILF2, Rbbp4, and Tcea1 were mainly observed in the nucleus of hepatocytes, instead of immune cells, which suggested the potential role of these 3 TFs in the regulation of NAFLD progression (Figure 4D and 4G). To verify these findings, we further evaluated the 3 TFs expression in clinical liver samples. The expression of Rbbp4, Tcea1, and ILF2 were significantly downregulated in significant hepatic-steatosis NAFLD patients compared to non-significant hepatic-steatosis controls (Figure 4H and 4I). Due to individual differences, decreased trend was observed in ILF2, although difference did not reach significance (Figure 4I).

### Rbbp4, Tcea1, and ILF2 regulate lipid metabolism related genes expression

**Figure 5:**
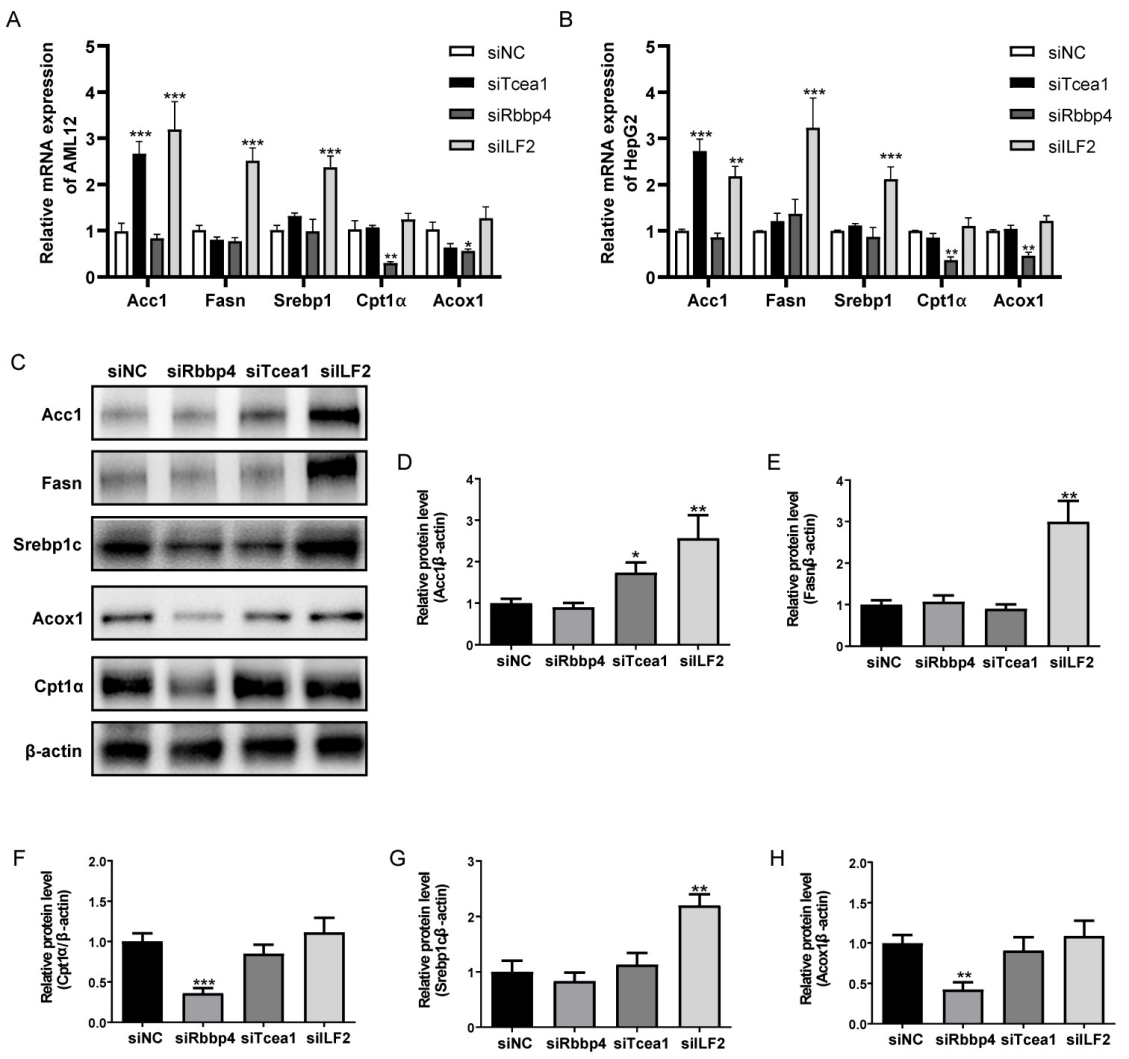
Tcea1, Rbbp4, and ILF2 regulated fatty acid synthesis or β-oxidation related gene expression. (A-B) RT-qPCR analysis of fatty acid synthesis related genes Acc1, Fasn, and Srebp1, and β-oxidation related genes Cpt1α, Acox1 expression in AML12 and HepG2 cells after Tcea1, Rbbp4, and ILF2 knockdown, respectively, compared with the siNC group. (C) Western blot analysis of Acc1, Fasn, Srebp1, Cpt1α, and Acox1 expression in AML12 cells after Tcea1, Rbbp4, and ILF2 knockdown, respectively. (D-H) Quantification of relative protein expression level. n=3 biological replicates. All data represent the mean ± SEM. Student's t test, * P < 0.05, ** P < 0.01, *** P < 0.001.

To investigate the role of Rbbp4, Tcea1, and ILF2 in cellular lipid metabolism, these 3 TFs were knocked down in AML12 and HepG2 hepatocytes by siRNA silencing strategy. 3 siRNA oligos targeting different mRNA regions were constructed for each gene. The siRNA oligo achieved more than 90% knockdown efficacy of mRNA expression compared to the siRNA control (Figure S3). The siRNA with the highest knockdown efficacy was used for further gene functional study in AML12 and HepG2 cells. Based on the potential interactions between Rbbp4, Tcea1, and ILF2 with key regulators in lipid metabolism (Figure 3C), we first evaluated the effects of Rbbp4, Tcea1, and ILF2 knockdown on the expression of key genes involved in lipid metabolism. Fatty acid synthesis genes Acc1, Fasn, Srebp1, and β-oxidation related genes Cpt1α, Acox1 were detected by qPCR and western blot analysis. Total RNA and proteins were extracted 48 h after siRNA transfection in AML12 and HepG2 cells. As shown in Figure 5A and 5B, the mRNA expression of Acc1 in Tcea1 knockdown cells was siginificantly incresed, and ILF2 downregulation significantly promoted Acc1, Fasn, and Srebp1 expression. In addition, the expression of Cpt1α and Acox1 expression was significantly decreased when Rbbp4 was downregulated. Protein expression detected by western blot in AML12 cells showed the same result (Figure 5C-5H). The results demonstrated that Tcea1 and ILF2 knockdown promoted fatty acid synthesis, and Rbbp4 knockdown inhibited fatty acid β-oxidation, which may lead to lipid accumulation in hepatocytes.

### Rbbp4, Tcea1, and ILF2 deficiency promotes lipid accumulation in hepatocytes

**Figure 6:**
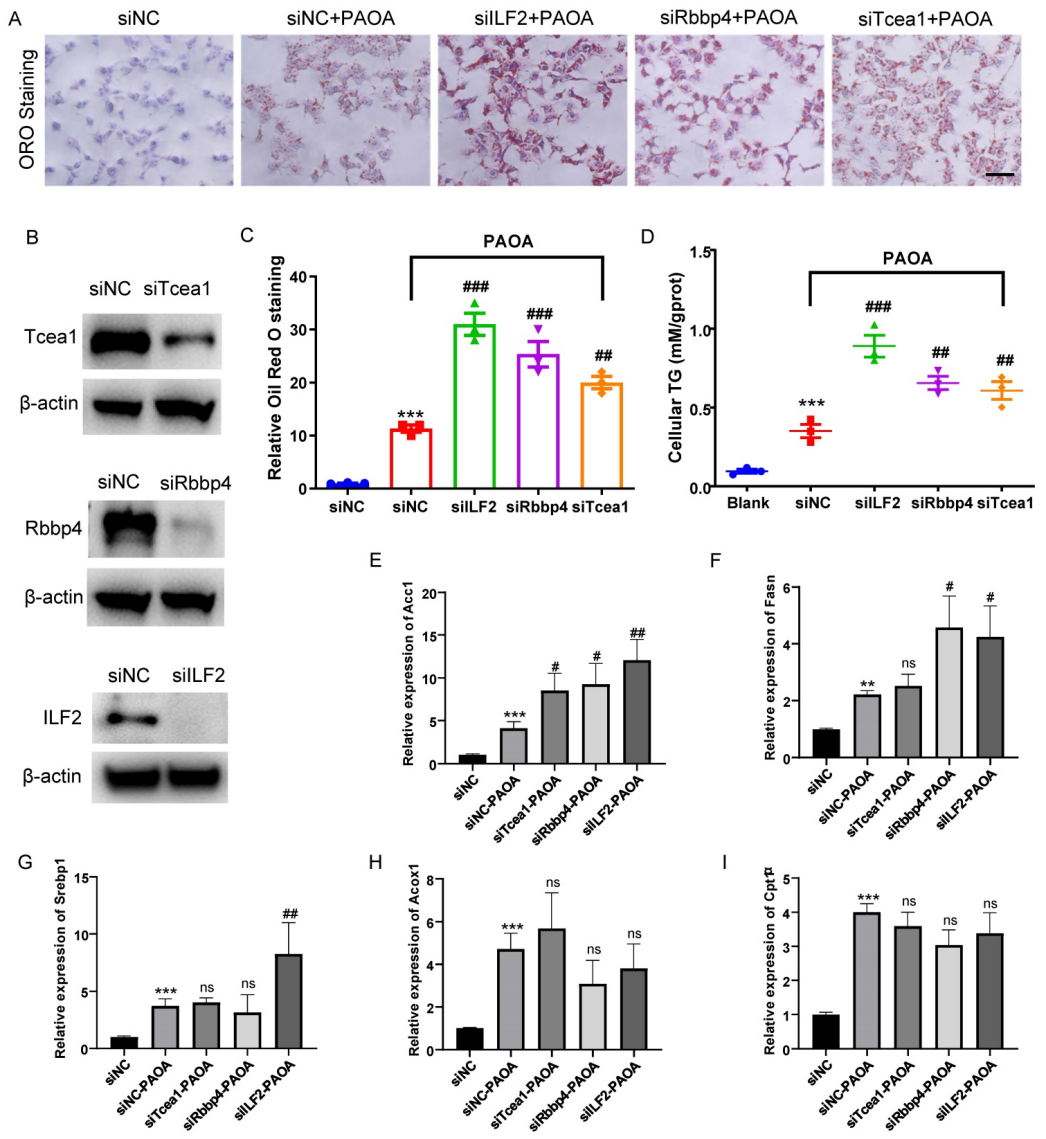
Tcea1, Rbbp4, and ILF2 deficiency promoted lipid accumulation of hepatocytes, respectively. (A) Representative images of Oil red O (ORO) staining of AML12 cells treated with or without PAOA for 24 h after Tcea1, Rbbp4, and ILF2 knockdown, respectively. Scale bar: 100 μm. (B) Western blot analysis of Tcea1, Rbbp4, and ILF2 expression in AML12 cells used for ORO staining. (C) Quantification of relative ORO positive area. (D) Measurement of cellular TG concentration in gene knockdown AML12 cells after PAOA treatment for 24 h. (E-I) RT-qPCR analysis of Acc1, Fasn, Srebp1, Cpt1α, and Acox1 gene expression in AML12 cells treated with or without PAOA after Tcea1, Rbbp4, and ILF2 knockdown, respectively. siNC represents cells transfected with negative control siRNA. n=3 biological replicates. All data represent the mean ± SEM. Student's t test, ** P < 0.01, *** P < 0.001 compared with the siNC group; ## P < 0.01, ### P < 0.001 compared with the siNC+PAOA group.

AML12 cells were transfected with siRNA for 48h and then treated with PAOA for another 24 h, and the control group was treated with corresponding amount of BSA. Protein expressions of Rbbp4, Tcea1, and ILF2 in AML12 cells were detected by western blot (Figure 6B). Through Oil red O staining and cellular TG concentration measurement, we observed that compared with the control, Rbbp4, Tcea1, and ILF2 knockdown significantly increased cellular lipid accumulation (Figure 6A ,6C and 6D). In addition, Rbbp4, Tcea1, and ILF2 deficiency significantly promoted basal levels of lipid contents in HepG2 cells (Figure S4).

We further detected the expression of above-mentioned genes in Tcea1, ILF2, and Rbbp4 knockdown cells under fatty acid excess conditions. AML12 cells with or without gene knockdown were treated with PAOA for 24 h. Cells transfected with control siRNA and treated with BSA were used as control. PAOA treatment significantly increased fatty acid synthesis and β-oxidation related gene expression compared with the control (Figure 6E-6I). After PAOA exposure, Acc1 mRNA expression increased notably in all 3 gene knockdown cells compared with cells transfected with control siRNA (Figure 6E). Fasn mRNA expression level in ILF2 and Rbbp4 knockdown cells was nearly two-fold higher than that in control cells after PAOA treatment (Figure 6F). In addition, ILF2 knockdown, rather than Teca1 and Rbbp4 knockdown, significantly increased Srebp1 expression compared with control cells (Figure 6G). However, no significant change were observed in Acox1 and Cpt1α expression in all 3 genes konckdown cells after PAOA treatment (Figure 6H and 6I).

### Rbbp4, Tcea1, and ILF2 play beneficial role in alleviating hepatocyte steatosis

**Figure 7:**
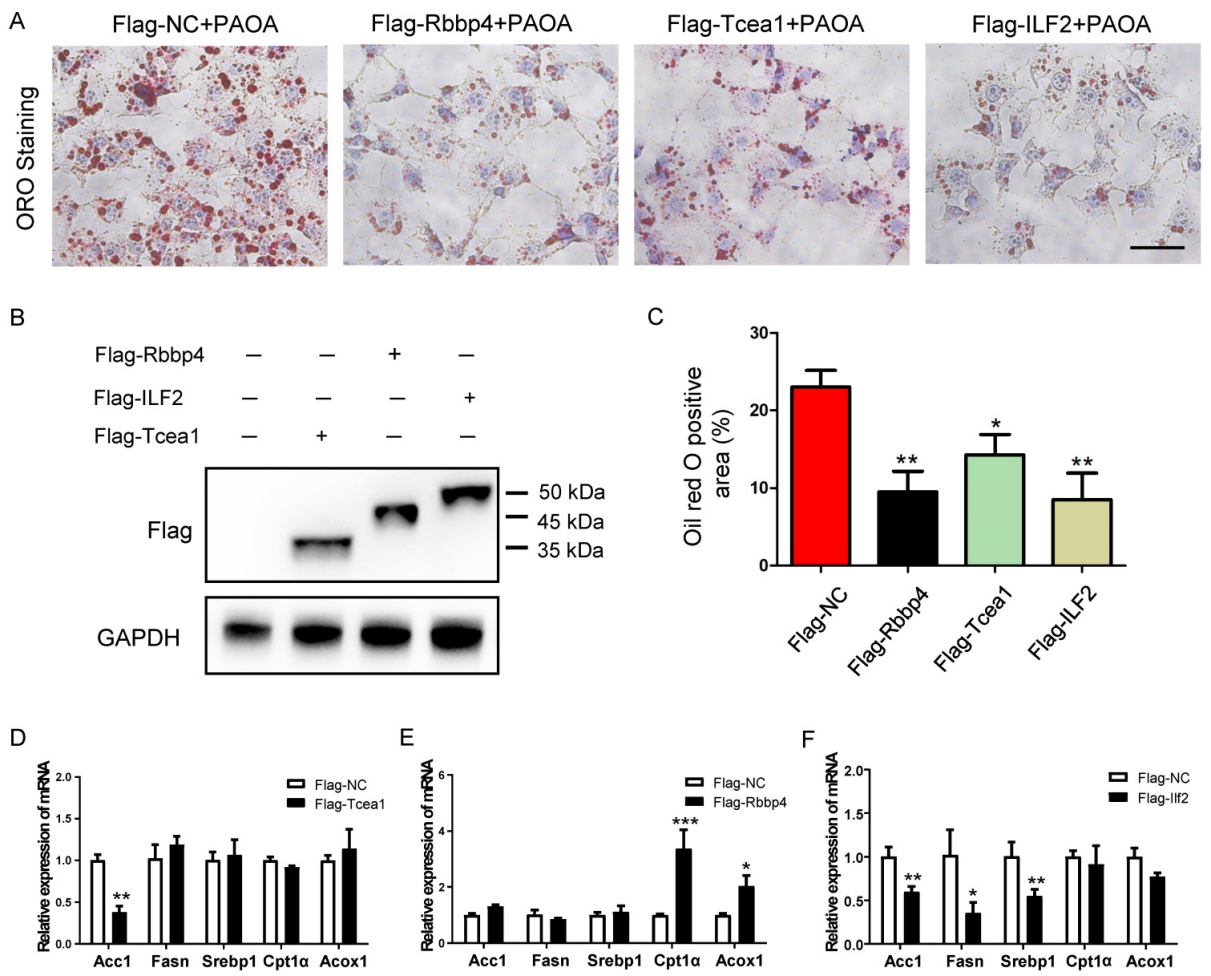
Overexpression of Tcea1, Rbbp4, and ILF2, respectively, in AML12 cells ameliorated hepatocyte steatosis. (A) Representative images of ORO staining of AML12 cells treated with PAOA after 48 h of transfection with Flag-NC, Flag-Tcea1, Flag-Rbbp4, and Flag- ILF2 plasmids, respectively. Scale bar: 100 μm. (B) Protein expression analysis of Flag-tagged Tcea1, Rbbp4, and ILF2 expression in AML12 cells after 48 h of transfection with corresponding plasmids. (C) Quantification of ORO positive area per field. n=3 biological replicates. (D-F) RT-qPCR analysis of Acc1, Fasn, Srebp1, Cpt1α, and Acox1 expression in AML12 cells after Tcea1, Rbbp4, and ILF2 overexpression, respectively, compared with the Flag-NC group. All data represent the mean ± SEM. Student's t test, * P < 0.05, ** P < 0.01.

To validate the role of the 3 TFs in regulating lipid metabolism, we generated mouse Rbbp4, Tcea1, and ILF2-overexpressing AML12 hepatocytes by transduction with a Flag-vector carrying corresponding gene (Figure 7B). After 48 h of transfection, cells were treated with PAOA for another 24 h. Compared to the Flag control, the Rbbp4, Tcea1, and ILF2-overexpression vectors significantly attenuated lipid accumulation in hepatocytes (Figure 7A and 7C). Overexpression of Tcea1 significantly inhibited the expression of Acc1, and Rbbp4 overexpression significantly promoted Cpt1α and Acox1 expression. In addition, the expression of Acc1, Fasn, and Srebp1 expression was significantly decreased when ILF2 was overexpressed. In summary, hepatocyte Rbbp4, Tcea1, and ILF2 regualted lipid metabolism genes expression, and exerted beneficial effects by suppressing lipid accumulation.

## Discussion

In the present study, we conducted the proteomic profiling of liver tissues of HFD-induced fatty liver mice by a label-free quantitative proteomics approach to elucidate the molecular mechanisms of fatty liver development. 2298 proteins were identified, of which 362 proteins were quantified as DEPs between the HFD and NCD groups. Further bioinformatics analysis of DEPs demonstrated that metabolism-related processes were mainly dysregulated by HFD. A subset of TFs having PPI network with key regulators in lipid metabolism was significantly enriched within DEPs. 7 TFs were further verified in vivo and in vitro, and 3 TFs were finally validated. Gene knockdown and overexpression experiments indicated that the 3 TFs played a suppressive role in hepatocyte steatosis through regulating the expression of genes related to lipid synthesis or oxidation. Thus, our study provides description of a potential role of Tcea1, Rbbp4, and ILF2 in the regulation of NAFLD for the first time.

High-fat diet induced metabolic disorders can lead to insulin resistance and hyperglycemia 23. Numerous proteomic studies of NALFD have shown that metabolism-related processes and pathways, including PPAR, AMPK, insulin signaling, are highly enriched through Gene ontology enrichment analysis, which is consistent with our results 15, 24. In addition, KEGG analysis also enriched other signaling pathways, such as HIF-1 signaling, steroid biosynthesis, and glucagon signaling. These pathways may also contribute to the development of NAFLD, and could be investigated in future studies. We also found that nearly 60% of DEPs were metabolism-related proteins, mainly enriched in fatty acid and organic acid metabolism processes. Furthermore, molecular function enrichment results show that more than 70% of DEPs have catalytic activity, including transferase activity, hydrolase activity, and oxidoreductase activity. Collectively, our results demonstrate that excessive lipid supply lead to the dysregulation of catalytic enzymes expression, which results in lipid metabolism disorders and promotes NAFLD.

A previous study has shown that during prolonged HFD, changes in global protein levels revealed a strong metabolic reprogramming toward lipid consumption and accumulation in response to excessive dietary lipid exposure, which suggests that prolonged HFD could coordinate metabolism in reponse to the lipid challenge 24. We noticed that 15% of DEPs were nucleic acid binding proteins, composed of RNA binding proteins and DNA binding proteins, playing crucial role in regulating gene expression. Upstream regulators were predicted based on the DEPs identified using the IPA analysis, we constructed a crucial interaction network consisting of protein-upstream regulator-biological processes, which revealed that SREBPs, PPAR-α, PPAR-γ, INSR, and ESRRA were key upstream regulators modulating lipid oxidation and lipid droplets formation. Of note, 4 of the 5 key regulators including SREBPs, PPAR-α, PPAR-γ, and ESRRA were TFs, suggesting aberrant activation of TFs might be a critical factor in regulating catalytic enzymes expression and driving liver metabolic disorders.

Sterol regulator element binding proteins (SREBPs) are a family of transcription factors involved in the biogenesis of cholesterol, fatty acids, and triglycerides 11, 25, 26. In the liver, SREBPs are involved in the pathogenesis of NAFLD, NASH, hepatitis and hepatocellular carcinoma 27. Nowadays, researches on SREBP inhibitory agents are being conducted. Inhibitors of SREBP processing can be helpful in reducing the risk of atherosclerosis, metabolic syndrome, and obesity, such as 27-HC and botulin 28, 29. A recent phase 2a clinical trial revealed that a DGTA2 inhibitor, which could reduce lipogenesis through downregulating SREBP1 expression, could enhance the therapeutic efficacy and overcome the side-effect of ACC inhibitors for NAFLD 30. The results demonstrate that TFs could be potential therapeutic targets for NAFLD.

Transcription factors are main factors that regulate gene expression, which are generally low-abundance proteins. Due to the technical difficulty of identifying low-aboundance regulatory proteins by mass spectrometry, few studies have sought to identify and characterize novel NAFLD-related TFs. In recent years, several nucleic acid binding proteins and TFs enrichment approaches have been established 31-33. With the advancement of mass spectrometry (MS)-based proteomics, substantial progress has been made in the quantitative measurement of TFs on a proteomic scale 33. Recently, *Liu et al.* have quantified hundreds of TFs with differential abundance of DNA-binding levels in the liver of obese mice compared with normal mice by using the TF response element-affinity enrichment method coupled with LC-MS/MS, which identified PU.1 as a novel TF in regulating NASH progression 34. In the present study, although only 11 DEPs were characterized as TFs by the whole proteome quantification, we observed their protein-protein interactions with key regulators and enzymes of lipid metabolism, suggesting their potential role in modulating NAFLD development. Finally, the expression of Rbbp4, TCEA1, and ILF2 were validated in both in vivo and in vitro models, especially in NAFLD clinical samples.

As demonstrated from our results, the identified candidate NAFLD-related TFs: Tcea1, Rbbp4, ILF2, Npm1, Ruvbl2, Npm1, Nucb1, Pc, Pura, Sra1, Preb, and Carhsp1 (Table 1) are found in the same clusters with already well-known NAFLD-related proteins (Figure 3F), enhancing their potential implication in NAFLD. Notably, Ruvbl2, Pc, Sra1, and Preb which were found upregulated in NAFLD liver tissue from our results, are literally confirmed for their regulation in promoting lipid accumulation of hepatocytes. However, our study provided a description of a role for Tcea1, Rbbp4, and ILF2 in the regulation of inhibiting lipid accumulation. The results demonstrate the high-confidence of our MS results, and also imply the complexity and heterogeneity of signaling networks interaction caused by metabolic disorders. To our knowledge, there are no any studies that have implicated these three proteins in the modulation of lipid metabolism and NAFLD.

Tcea1 is a transcript elongation factor that stimulates RNA cleavage by RNA polymerase II by allowing backtracked enzymes to resume transcription elongation 35. In human cells, Tcea1 affects transcript elongation and cell viability 36. Recent studies have unveiled the function of Tcea1 during stress responses, which demonstrated that tcea1 was an essential component for thermal adaptation in plants 37, preventing transcription stress 38 and oxidative stress 39. Herein, we observed that Tcea1 was also essential for fatty acid excess adaptation in hepatocytes. However, little is known about its regulation during stress responses, especially in pathological states. It has been reported that the expression of Tcea1 could be regulated by YEATS4, a NAFLD-related proteins, in HCC cell lines 40, 41. We therefore speculate that the downregulation of Tcea1 in NAFLD may be regulated by YEATS4, which should be investigate in future study.

Rbbp4 is a histone-binding subunit that regulate chromatin metabolism. As a core component of several complexes, Rbbp4 plays important role in histone modification, chromatin assembly and remodeling, which can regulate gene transcription, cell cycle, and proliferation 42. Moreover, RBBP4 has gained attention for its potential involvement in the mechanism of tumorigenesis, such as liver cancer 43, lung adenocarcinoma 44 and acute myeloid leukemia 45. It has been reported that Rbbp4, a subunit of NURF complex, is involved in the regulation of lipid droplet size by transcriptional repression of target genes 46. In the present study, we observed the protection role of rbbp4 in hepatocyte steatosis through promoting fatty acid β-oxidation related gene expression. However, the mechanism of its regulation in pathological states have not been reported.

ILF2 encodes NF45, the regulatory subunit of NF90/NF110 complexes that are involved in mitotic control and various aspects of RNA metabolism, including transcription, RNA transport, mRNA stability, and translation 47, 48. Depletion of the NF45 subunit is accompanied by a dramatic decrease in the levels of NF90 and NF110. Likewise, depletion of NF90 greatly reduces the level of NF45. NF45 is post-transcriptionally regulated by NF90, which is unstable and degraded in the absence of NF90. Beyond interacting with its known binding partners NF90 and NF110, ILF2 also interacts with numerous RNA-binding proteins (RBPs), including YB-1, NPM1, and various heterogeneous nuclear ribonucleoproteins (hnRNPs), which are directly involved in DNA repair and genome stability maintenance. However, the role of ILF2 in the regulation of lipid metabolism/NAFLD has not been reported. A recent study has demonstrated that YB-1, a ILF2 interacting protein, regulates liver lipid metabolism by regulating the Wnt/β-catenin signaling pathway 49, which implied the possibility of ILF2 in the regulation of NAFLD. Thus, this study reports that ILF2 is a potential positive modulator of lipid metabolism by inhibiting fatty acid biosynthesis genes expression.

However, the role of these 3 TFs in liver and NAFLD is still unknown. To better understand the functions of Rbbp4, TCEA1, and ILF2 in lipogenesis, we studied the effects of gene knockdown in hepatocytes on lipid accumulation and the expression of genes involved in adipogenesis and β-oxidation. We found that the downregulation of these 3 TFs significantly increased lipid accumulation in hepatocytes, and the phenotype could be rescued when they were overexpressed. In addition, TCEA1 and ILF2 mainly affected the expression of fatty acid synthesis-related genes, and several β-oxidation-related genes were downregulated after RBBP4 knockdown. However, the above-mentioned genes expression patterns were different under excessive lipid exposure, which indicated that in the context of NAFLD, diverse mechanisms might underlie the lipid accumulation reduction effect of the 3 TFs. The present study has limitations, one is lack of verification of Tcea1, Rbbp4, and ILF2 function in vivo by gene knockout/overexpression mice, another is lack of target gene screening for Tcea1, Rbbp4, and ILF2 which will be explored in future studies.

In summary, our study provides a comprehensive analysis of protein expression patterns and biological processes perturbations involved in the development of NAFLD by quantitative proteomics. Of particular translational interest is the finding that we identified three novel NAFLD-related TFs, Tcea1, Rbbp4, and ILF2, which were validated in the NAFLD model mice and clinical liver samples. Knockdown of the 3 genes promoted hepatocyte steatosis by dysregulating the expression of genes involved in adipogenesis and β-oxidation, respectively. These findings proposed novel lipid metabolism-related TFs, which might have potential roles in preventing NAFLD.

## Supplementary Material

Supplementary methods, figures and table.

## Figures and Tables

**Table 1:** Differentially expressed transcription factors quantified by LC-MS/MS.

Protein IDs	Gene names	Protein names	Unique peptides	Sequence coverage [%]	Mol. weight [kDa]	Fold change (HFD/NC)	p value
P10711	Tcea1	Transcription elongation factor A protein 1	2	9	33.88	0.01	3.77E-02
Q9WTM5	Ruvbl2	RuvB-like 2	6	14.5	51.112	0.34	1.44E-02
Q61937	Npm1	Nucleophosmin	9	31.8	32.56	0.65	1.62E-02
Q02819	Nucb1	Nucleobindin-1	12	32.9	53.408	1.51	2.06E-02
Q05920	Pc	Pyruvate carboxylase	54	48.8	129.68	1.53	9.08E-05
P42669	Pura	Transcriptional activator protein Pur-alpha	5	18.7	34.883	2.94	2.63E-02
Q80VJ2	Sra1	Steroid receptor RNA activator 1	2	9.1	25.557	3.50	1.40E-02
Q9WUQ2	Preb	Prolactin regulatory element-binding protein	7	22.1	45.437	3.72	2.41E-02
Q9CR86	Carhsp1	Calcium-regulated heat stable protein 1	4	26.4	16.062	11.78	1.95E-02
Q60972	Rbbp4	Histone-binding protein RBBP4	3	19.1	47.655	0.47	1.86E-02
Q9CXY6	Ilf2	Interleukin enhancer-binding factor 2	4	12.1	43.062	0.38	4.02E-03

## Abbreviations

NAFLD: Nonalcoholic fatty liver disease
TFs: transcription factors
HFD: High fat diet
NCD: Normal chow diet
FASN: fatty acid synthase
ACC: acetyl-CoA
Cpt1α: carnitine palmitoyltransferase-1α
SREBP1: sterol regulatory element-binding transcription factor 1
Tcea1: transcription elongation factor A protein 1
Rbbp4: histone-binding protein RBBP4
ILF2: interleukin enhancer-binding factor 2
DEPs: differential expressed proteins
IPA: Ingenuity Pathway Analysis
GTT: glucose tolerance test
ITT: insulin tolerance test
PAOA: palmitic acid/oleic acid
