# Supplementary material for: Quantitative proteomics of HFD-induced fatty liver uncovers novel transcription factors of lipid metabolism
Source: Int J Biol Sci. 2022 May 1;18(8):3298–312. doi: 10.7150/ijbs.71431 (PMC9134917; doi:10.7150/ijbs.71431)
Supplement: Supplementary file 1 — Supplementary methods, figures and table. [file ijbsv18p3298s1.pdf]

Supplemental materials

Quantitative proteomics of HFD-induced fatty liver uncovers novel transcription factors of lipid metabolism

Shang Zhi[1,2]*, Zhang Congcong[1]*, Gao Zhiling[3]*, Qian Yihan[1,2], Xin Yijing[1], Liu Guanjie[1], Wang Fang[1], Sun Xuehua[1], Li Hongjie[4†], Kong Xiaoni[1†], Gao Yueqiu[1,2†].

1 Central Laboratory, Department of Liver Diseases, ShuGuang Hospital Affiliated to Shanghai University of Chinese Traditional Medicine, Shanghai, China

2 Institute of Infectious Disease, ShuGuang Hospital Affiliated to Shanghai University of Chinese Traditional Medicine, Shanghai, China

3 Department of Ultrosound, Shuguang Hospital Affiliated to Shanghai University of Chinese Traditional Medicine, Shanghai, China

4 Department of General Surgery, Shanghai Ninth People' Hospital, Shanghai Jiao Tong University School of medicine, Shanghai, China

Authorship note: * These authors contributed equally to this study.

†Corresponding authors: Kong Xiaoni, PhD, Central Laboratory, ShuGuang Hospital Affiliated to Shanghai University of Chinese Traditional Medicine, 528 Zhangheng Road, Shanghai, China, 201203. Telephone: +86 21 20256838. E-mail: xiaoni-kong@126.com; Or Gao Yueqiu, MD, gaoyueqiu@hotmail.com; Or Li Hongjie, MD, shanghaileester@gmail.com

Supplemental methods

Glucose tolerance tests and insulin tolerance tests

Glucose tolerance was monitored by glucose tolerance tests (GTT) 10 days before sacrificing the mice. One week later, the same mice were used for insulin tolerance tests (ITT). For GTT, mice were fasted for 12 h. After measuring the baseline blood glucose level via a tail nick using a glucometer, 1.5 g/kg glucose was administered via intraperitoneal injection, and glucose levels were measured at 0, 30, 60, and 120 min after the glucose injection. For ITT, mice fasted for 6 h were injected intraperitoneally with recombinant human insulin at a dose of 1 U/kg, and their blood glucose concentrations were determined at 0, 15, 30, 60, and 120 min after the insulin injection.

Primary hepatocytes isolation, cell culture, and Oil red O staining

Primary hepatocytes were isolated from mice fed with normal chow diet. Briefly, after anesthesia, mice were perfused with a buffer solution lacking $Ca2^{+}$ and $Mg2^{+}$ through the portal vein, and then perfused with 0.05% type IV collagenase. After digestion, the liver was excised, minced, and filtered through a 70 μm filter. Then, hepatocytes were separated by centrifugation at 50 × g for 2 min twice. The obtained hepatocytes were resuspended in DMEM supplemented with 10% FBS and 1% penicillin streptomycin, and seeded in six-well plates. 0.5mM of palmitate acid (PA) and 1mM oil acid (OA) was added to the medium for 24 h to establish the in vitro model of lipid accumulation in hepatocytes.

AML12 and HepG2 cells were obtained from the Type Culture Collection of the Chinese Academy of Science and were cultured in DMEM/F12 supplemented with 10% FBS and 1% penicillin streptomycin. Cell cryopreservation using serum-free cell freezing medium according to manufacturer's instructions (UUbio, Suzhou, China). The cells were seeded in 12-well plates at 1×10^{5} cells/well. 0.5mM palmitic acid (PA) and 1mM oil acid (OA) were added to the medium for 24 h to establish the in vitro model of lipid accumulation in AML12 cells. The cells were fixed with 4% paraformaldehyde

for 20 min, and then stained with 60% Oil Red O working solution for 1 min to examine intracellular lipid accumulation.

RNA extraction and RT-qPCR

Total RNA was extracted from mouse livers and hepatocytes using Total RNA extraction kit according to manufacturer's instructions (UUbio, Suzhou, China). 1 μg of total RNA was reverse-transcribed to cDNA using the HiScript II 1st Strand Cdna Synthesis Kit (Vazyme, Nanjing, China). RT-qPCR was performed using the SYBR Green PCR kit (Vazyme, Nanjing, China) on the Light Cycler 480 realtime PCR system (Roche, Switzerland). Gene expression was normalized to the expression of housekeeping gene Gapdh. The primers are listed in Table S1. The relative expression of mRNA was calculated using the $2^{-\Delta\Delta Ct}$ method.

Table S1. siRNA and primer sequences used are listed below.

Gene name	siRNA sequences (5'-3')	
Tcea1-1	CGAGAUACAUAUGUUUCAU	
Tcea1-2	GUACGAAGUAGGAUAUCAA	
Tcea1-3	GUAGUGCUGAUGAACCAAU	
Ilf2-1	GCAGGUAGGAUCAUAUAAA	
Ilf2-2	CCCUAGAACAACAGGAUAU	
Ilf2-3	GCCTTGCTGAAGAGGAATCA	
Rbbp4-1	CACCAGAAUUGUUGUUUAU	
Rbbp4-2	GCUGAAGUGAAUUGCUUAU	
Rbbp4-3	CCUGCAUCAUUGCAACAAA	
	Forword primers (5'-3')	Reverse primers (5'-3')
Tcea1	GAGAAGAAAGTAGTTCCAGCAGC	GCCCGAGGAAAAGATGAAACAT
ILF2	CCTGGGGAACAAAGTCGTGG	TGAGAATTTTCACCGTAGCATCA
Rbbp4	GACGACGCAGTGGAAGAAC	CTGGGCAGTTAAGCTGGGC
Acc1	ATGGGCGGAATGGTCTCTTTC	TGGGGACCTTGTCTTCATCAT
Fasn	GGAGGTGGTGATAGCCGGTAT	TGGGTAATCCATAGAGCCCAG
Acox1	TAACTTCCTCACTCGAAGCCA	AGTTCCATGACCCATCTCTGTC
Cpt1α	CTCCGCCTGAGCCATGAAG	CACCAGTGATGATGCCATTCT
Srebp1	GCAGCCACCATCTAGCCTG	CAGCAGTGAGTCTGCCTTGAT
Gapdh	AGGTCGGTGTGAACGGATTTG	TGTAGACCATGTAGTTGAGGTCA

Figure legends

Figure S1. HFD mice displayed enhanced glucose intolerance and insulin resistance compared to NCD mice. (A) glucose tolerance test (GTT) on HFD and NCD-fed mice. (B) insulin tolerance test (ITT) on HFD and NCD-fed mice. N=7 biological replicates. All data represent the mean ± SEM. Student's t test, *** $p < 0.001$.

Figure S2. (A) Biological processes enrichment of DEPs. (B) Weight gene analysis of DEPs.

Figure S3. (A-C) RT-qPCR showing knockdown efficiency of Tcea1, Rbbp4, and ILF2 in AML12 cells transfected with corresponding siRNA for 48 h. (D-F) RT-qPCR showing knockdown efficiency of Tcea1, Rbbp4, and ILF2 in HepG2 cells transfected with corresponding siRNA for 48 h.

Figure S4. Tcea1, Rbbp4, and ILF2 deficiency promoted lipid accumulation of HepG2 cells, respectively. (A) Representative images of Oil red O (ORO) staining of HepG2 cells after Tcea1, Rbbp4, and ILF2 knockdown, respectively. Scale bar: 100 μm. (C) Quantification of relative ORO positive area. All data represent the mean ± SEM. Student's t test, ** $P < 0.01$, *** $P < 0.001$

Figure S1

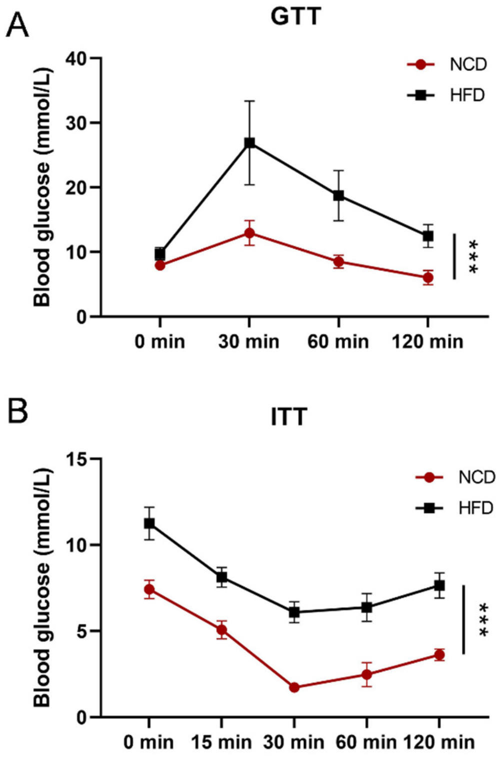
A
GTT
Blood glucose (mmol/L)
40
30
20
10
0
NCD
HFD
***
0 min
30 min
60 min
120 min
B
ITT
Blood glucose (mmol/L)
15
10
5
0
NCD
HFD
***
0 min
15 min
30 min
60 min
120 min

Figure S2

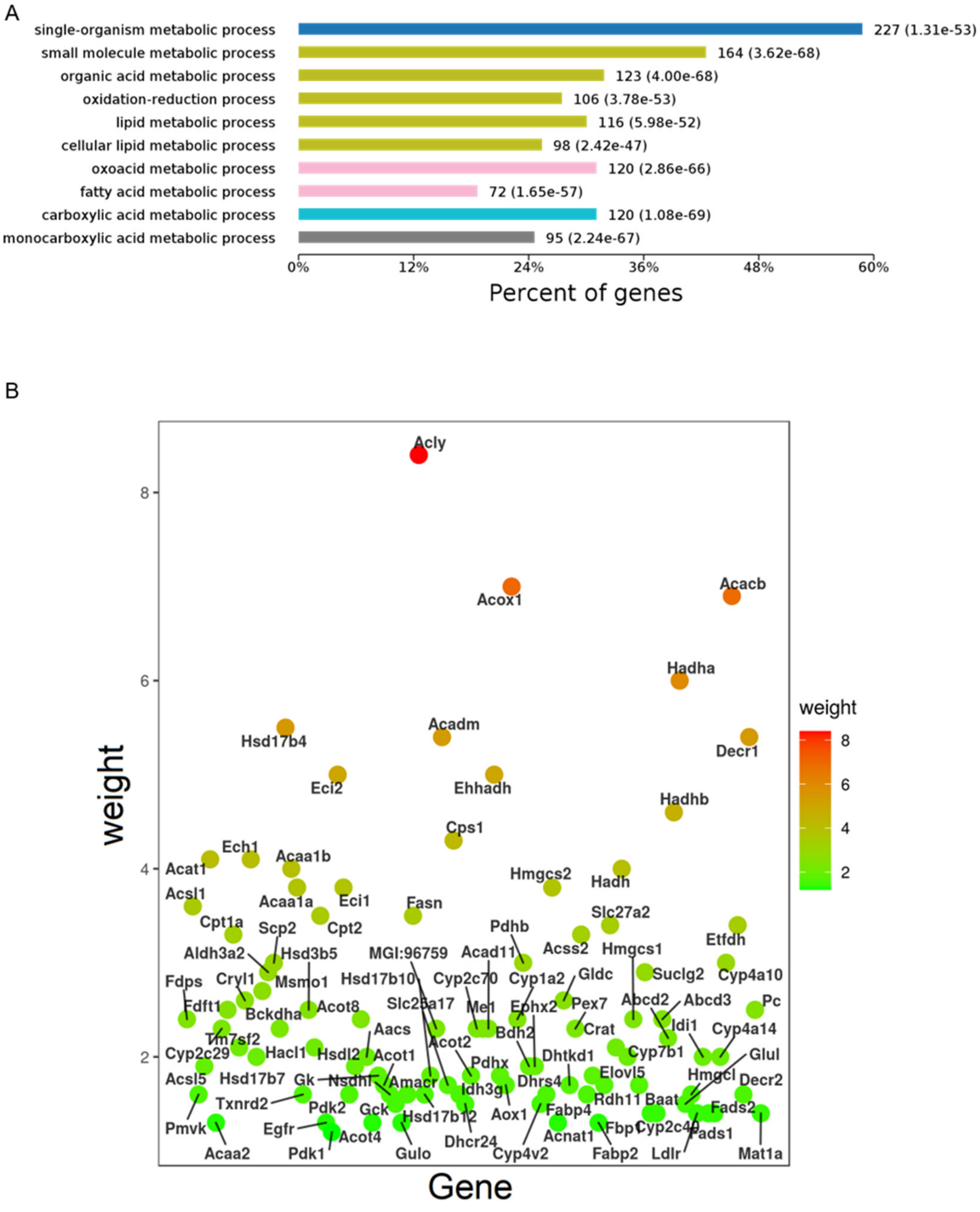
A
single-organism metabolic process
small molecule metabolic process
organic acid metabolic process
oxidation-reduction process
lipid metabolic process
cellular lipid metabolic process
oxoacid metabolic process
fatty acid metabolic process
carboxylic acid metabolic process
monocarboxylic acid metabolic process
227 (1.31e-53)
164 (3.62e-68)
123 (4.00e-68)
106 (3.78e-53)
116 (5.98e-52)
98 (2.42e-47)
120 (2.86e-66)
72 (1.65e-57)
120 (1.08e-69)
95 (2.24e-67)
0%
12%
24%
36%
48%
60%
Percent of genes
B
weight
Gene
8
6
4
2
Acly
Acox1
Acacb
Hadha
Hsd17b4
Acadm
Decr1
Eci2
Ehhadh
Hadhb
Cps1
Ech1
Acaa1b
Acat1
Hmgcs2
Hadh
Acsl1
Acaa1a
Eci1
Fasn
Slc27a2
Cpt1a
Scp2
Cpt2
Pdhb
Acss2
Hmgcs1
Etfdh
Aldh3a2
Hsd3b5
MGI:96759
Acad11
Fdps
Cryl1
Msmo1
Hsd17b10
Cyp2c70
Cyp1a2
Gldc
Suclg2
Cyp4a10
Fdft1
Bckdha
Acot8
Slc25a17
Me1
Ephx2
Pex7
Abcd2
Abcd3
Pc
Tm7sf2
Aacs
Bdh2
Crat
Idi1
Cyp4a14
Cyp2c29
Hacl1
Hsdl2
Acot1
Acot2
Pdhx
Dhtkd1
Cyp7b1
Glul
Acsl5
Hsd17b7
Gk
Nsdhl
Amacr
Idh3g
Dhrs4
Elovl5
Hmgcl
Decr2
Txnrd2
Pdk2
Gck
Hsd17b12
Aox1
Fabp4
Rdh11
Baat
Fads2
Pmvk
Egfr
Acot4
Dhcr24
Acnat1
Fbp1
Cyp2c40
Fads1
Acaa2
Pdk1
Gulo
Cyp4v2
Fabp2
Ldlr
Mat1a

Figure S3

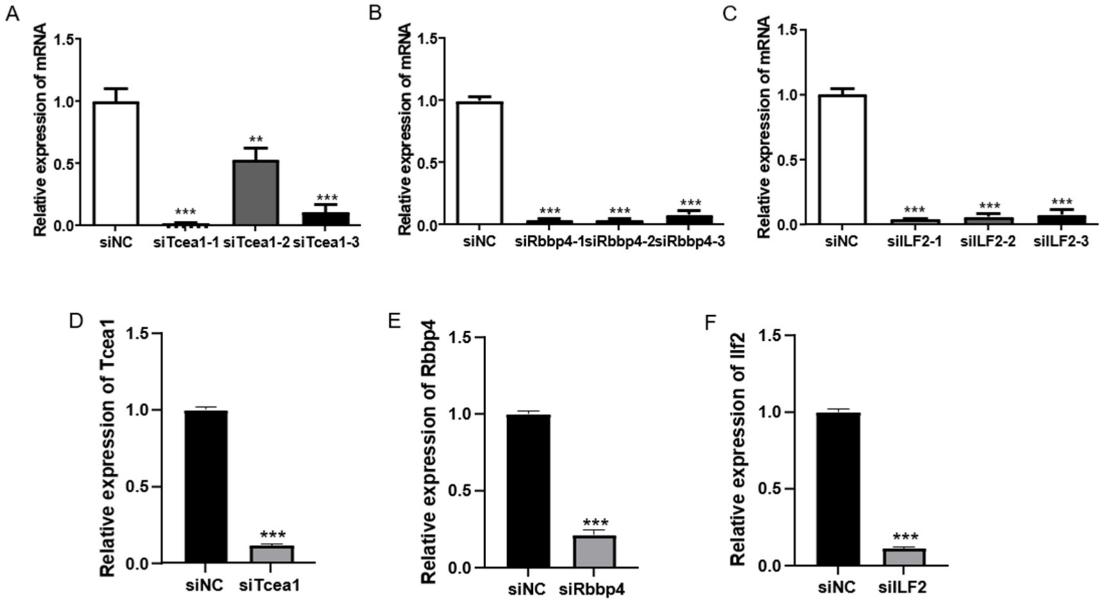
A
Relative expression of mRNA
1.5
1.0
0.5
0.0
**
***
***
siNC
siTcea1-1
siTcea1-2
siTcea1-3
B
Relative expression of mRNA
1.5
1.0
0.5
0.0
***
***
***
siNC
siRbbp4-1
siRbbp4-2
siRbbp4-3
C
Relative expression of mRNA
1.5
1.0
0.5
0.0
***
***
***
siNC
siILF2-1
siILF2-2
siILF2-3
D
Relative expression of Tcea1
1.5
1.0
0.5
0.0
***
siNC
siTcea1
E
Relative expression of Rbbp4
1.5
1.0
0.5
0.0
***
siNC
siRbbp4
F
Relative expression of Ilf2
1.5
1.0
0.5
0.0
***
siNC
siILF2

Figure S4

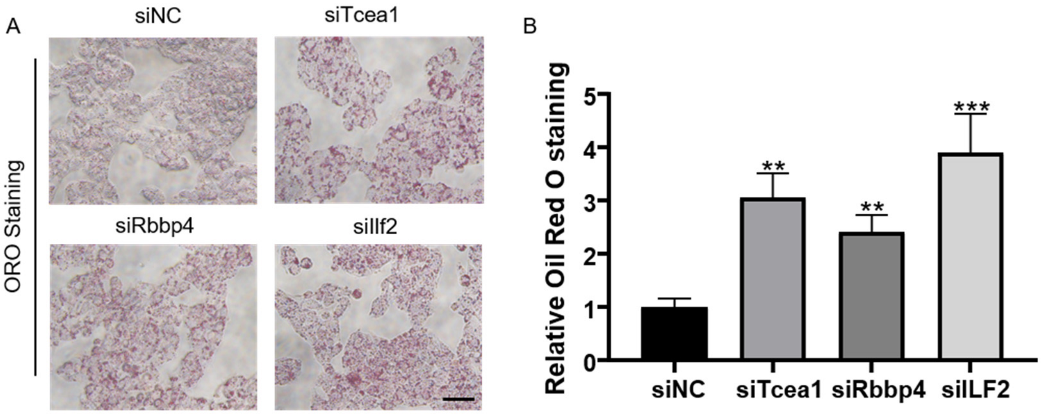
A
siNC
siTcea1
siRbbp4
siIlf2
ORO Staining
B
Relative Oil Red O staining
0
1
2
3
4
5
**
**
***
siNC
siTcea1
siRbbp4
siILF2
